# Trait anxiety is associated with amygdala expectation and caloric taste receipt response across eating disorders

**DOI:** 10.1038/s41386-022-01440-z

**Published:** 2022-09-13

**Authors:** Guido K. W. Frank, Megan E. Shott, Tamara Pryor, Skylar Swindle, Tyler Nguyen, Joel Stoddard

**Affiliations:** 1 Department of Psychiatry, University of California at San Diego, San Diego, CA USA; 2 ED Care, Denver, CO USA; 3 Department of Psychiatry, University of Colorado, Anschutz Medical Campus, Aurora, CO USA

**Keywords:** Psychiatric disorders, Medical research

## Abstract

Anxious traits are elevated in eating disorders (EDs), are considered risk factors for ED development, and trait anxiety has been linked to ED psychopathology. How trait anxiety relates to ED neurobiology is not well understood. In this study 197 individuals across the ED spectrum (anorexia nervosa *n* = 91; other specified EDs *n* = 34; bulimia nervosa *n* = 56; binge ED *n* = 16), and 120 healthy controls were assessed for anxious traits and learned to expect and receive caloric or neutral taste stimuli during brain imaging. Amygdala sucrose expectation response differed across groups (Wilk’s lambda = 0.945, *p* = 0.023), and was higher on the left in anorexia nervosa compared to healthy controls (*p* = 0.002). Expected sucrose receipt response across taste reward regions was not different between groups. In the ED sample, trait anxiety negatively moderated the relationship between amygdala expectation and right dorsal (*p* = 0.0062) and ventral (*p* = 0.0046) anterior insula receipt response. A subgroup analysis showed similar results for anorexia nervosa, and partially in bulimia nervosa. Across EDs, appetitive motivation correlated positively with bilateral orbitofrontal cortex, caudate head, and ventral striatal sucrose receipt response (*r* = 0.215 to 0.179, *p* = 0.002 to 0.012). Across the study sample, trait anxiety showed an inverted-U-shaped relationship with right (*r* = 0.147, *p* = 0.034) and left (*r* = 0.162, *p* = 0.016) amygdala expectation response. Amygdala sucrose expectation response is elevated in anorexia nervosa, correlates with sucrose receipt response, and this relationship is negatively moderated by trait anxiety across EDs. Trait anxiety may have an important role in how expectation drives taste stimulus receipt brain response and perhaps food approach in individuals with EDs.

## Introduction

Eating disorders (EDs) are severe psychiatric disorders with complex bio-psycho-social etiology [1]. Individuals with anorexia nervosa (AN) are underweight and may intermittently binge-eat or purge, individuals with bulimia nervosa (BN) tend to be at normal to high weight and regularly binge-eat and purge, while individuals with binge-eating disorder (BED) regularly binge-eat without compensatory behaviors [2]. The Other Specified Feeding and Eating Disorders (OSFED) category encompasses EDs that do not meet full criteria for AN, BN or BED. While individuals with EDs present with a range of behaviors from food restriction to overeating, they typically share high body dissatisfaction and drive for thinness. Other transdiagnostic behaviors that are thought to contribute to the often-chronic course of EDs include difficulty tolerating strong emotional states, anxiety, sadness or anger [3, 4].

Various studies have suggested that anxious traits such as trait anxiety and harm avoidance are potential risk factors for EDs [5–7]. Individuals with those traits experience negative emotions including fears, worries, and anxiety across many situations and tend to perceive environmental stimuli as threatening [8–10]. Trait anxiety has been associated with ED psychopathology previously [11–13]. For instance, one study found that trait anxiety was related to low self-confidence and avoidance of social eating across EDs [14]. Furthermore, anxiety moderated the relationship between body dissatisfaction and disordered eating, suggesting that non-specific anxiety contributes to ED behaviors and severity [15]. Brain research that focused on trait anxiety irrespective of EDs found this temperament trait to be associated with amygdala activation, implicating the amygdala as potentially important in ED neurobiology [16–18]. In fact, a few studies have found elevated or reduced amygdala response in groups with EDs in response to body image, taste or emotional conflict tasks [19–21].

Neurobiological studies have repeatedly suggested that brain reward circuits are part of ED pathophysiology [22]. Recent results from our group across a transdiagnostic sample of individuals with EDs indicated that brain response in the motivational salience brain circuitry is related to body mass index (BMI) and striatal-hypothalamic food control pathways, reinforcing ED behaviors [23]. That study supported the hypothesis that extremes of food restriction or overeating alter dopamine related brain response, and anxious conditioning to food intake may recruit those circuits to engage in fearful avoidance as opposed to food approach [24, 25].

How trait anxiety and the neurobiology of reward circuits interact across EDs is not well understood but could have important implications on food intake behaviors [26, 27]. The majority of research that investigated fears and anxiety in individuals with EDs has used food pictures, but those studies had limited success in identifying underlying mechanisms of EDs [28]. Applying caloric and non-caloric taste stimuli during brain imaging, however, better relates to actual eating and thus fear of caloric food intake, engages well defined neural pathways and can be used to integrate fear and reward circuitry [23]. Neurotransmitter receptor studies repeatedly indicated relationships between receptor binding and measures for anxious traits in individuals with EDs [29]. Anticipatory anxiety and arousal have been associated with meal anxiety in a study that investigated interoception in AN using a sympathetic agonist, and that study highlighted anxious traits as important for altered interoception and anxious anticipation in the disorder [30]. That study suggested that non-specific anxious traits directly affect ED-related psychopathology.

Studies in the past showed that neural response to stimulus expectation and receipt are closely linked [31, 32]. This led us to hypothesize that brain response to expecting a caloric stimulus might drive activation in response to receipt of that stimulus. Furthermore, we hypothesized that trait anxiety would drive the interaction between expectation and caloric stimulus response. Such a finding would be relevant for the clinical care of individuals with EDs as it could indicate that treatments focusing on anxious traits could be useful for ameliorating food-related anxiety in individuals with EDs and normalizing eating behavior [33].

Here we investigated the above described transdiagnostic study sample across the ED spectrum [23] to test response to caloric stimulus (sucrose) expectation and expected receipt, both contrasted against non-caloric taste stimulus, and the effects of anxious traits. We hypothesized that elevated trait anxiety in individuals with EDs would be associated with brain response to caloric taste stimulus expectation and receipt. The amygdala is a brain region central to expectation, vigilance, anxiety and threat [34, 35]. We expected that the ED sample would show elevated amygdala response to *expectation* of a high caloric sucrose solution stimulus compared to the non-caloric stimulus and that expectation response would negatively bias and reduce response in brain reward regions during expected taste *receipt*, which could interfere with food intake [36].

## Methods and materials

### Participants

The Colorado Multiple Institutional Review Board approved the study. All participants provided written informed consent. Procedures including recruitment and sample size were conducted according to the approved and funded study (NIMH-R01MH103436). We recruited 197 women with an ED: 69 AN restricting subtype, 22 AN binge-eating/purging subtype, 17 OSFED atypical AN subtype, 17 OSFED purging disorder subtype, 56 BN, 3 OSFED binge-eating subtype, and 13 binge ED (BED). To increase power for comparison with HC, we combined restrictive and binge-eating/purging AN subgroups (AN, severe food restriction), OSFED Atypical AN and Purging Disorder subgroups (OSFEDr, intermediate restrictive eating, normal BMI), as well as OSFED binge-eating and BED groups (BED, loss of control eating, elevated BMI). ED participants were recruited from ED partial hospitalization specialty care (EDCare Denver or Children’s Hospital Colorado) within the first 2 weeks of treatment, to mitigate effects of acute starvation or dehydration [37]. Following NIMH’s Research Domain Criteria (RDoC) instructions, we recruited “any interested ED patient” who was admitted to treatment. In addition, we recruited 120 healthy control women (HC) without lifetime psychopathology through local advertisements.

Participants were right-handed without history of head trauma, neurological disease, major medical illness, bipolar disorder, psychosis, or current (past 3 months) substance use disorder. HC were studied during the first 10 days of the menstrual cycle to reduce hormonal effects. For EDs, treatment stage was the primary variable we controlled for, but we recorded days from last menstrual cycle as a proxy to test for effects of hormonal variation.

### Assessments

Psychiatric diagnoses were assessed using the Structured Clinical Interview for DSM-5 (doctoral-level interviewer) [38]. Participants completed the State-Trait Anxiety Inventory [8], Temperament and Character Inventory for Harm Avoidance [39], Behavior Inhibition/Fear-Fight-Freeze/Behavior Approach System (BIS/FFFS/BAS) for Flight-Fight-Freeze System (FFFS), BIS Anxiety, BAS-Reward Responsiveness, BAS Drive and BAS-Fun Seeking [40], Eating Disorder Inventory–3 for Drive for Thinness (intense fear of weight gain), Bulimia (tendency to engage in binge eating), and Body Dissatisfaction (discontentment with size of body regions) [41], Beck Depression Inventory-II [42], and participants blindly rated sugar solutions for sweetness and pleasantness using a 9-point Likert scale.

### Brain imaging methods

#### Functional magnetic resonance imaging (fMRI)

Between 0700 and 0900 h, ED participants ate their meal-plan breakfast and healthy controls ate a quality- and calorie-matched breakfast. FMRI of the brain was performed between 0800 and 0900 h on either a 3T GE Signa or Siemens Skyra 3T scanner (see Supplementary Material).

#### Taste reward task

The design (Supplementary Material) was adapted from O’Doherty et al. [43]. Participants learned to associate three unconditioned taste stimuli (US: 1 molar [M] sucrose solution, no solution, or artificial saliva) with paired conditioned visual stimuli (CS). Each CS was probabilistically associated with its US such that 80% of sucrose and no solution CS trials were followed by sucrose or no solution, respectively. CS and US for expectation and expected receipt of sucrose or artificial saliva were analyzed. For this study, only correct expectation–receipt trials were analyzed.

#### fMRI analysis

Image preprocessing and analysis were performed using SPM12 (http://www.fil.ion.ucl.ac.uk/spm/software/spm12/). Images were realigned to the first volume, normalized to the Montreal Neurological Institute template, smoothed at 6 mm full-width-at-half-maximum Gaussian kernel. Data were preprocessed with slice time correction and modeled with a hemodynamic response convolved function using the general linear model, including temporal and dispersion derivatives. A 128-s high-pass filter (removing low-frequency BOLD signal fluctuations), 6 motion parameters (as first-level analysis regressors), and SPM’s FAST (pre-whitening attenuation of autocorrelation effects) were applied [44].

#### Taste expectation and receipt analysis

We developed first-level models to predict the response in each voxel as a function of the following conditions: (1) sucrose expectation: trials with CS predicting sucrose receipt contrasted against trials with CS predicting artificial saliva (non-caloric taste stimulus); (2) expected sucrose receipt: trials with expected US caloric sucrose receipt contrasted against trials with expected US non-caloric artificial saliva receipt.

#### Region of interest (ROI) data extraction

We extracted beta values from predefined regions of interest bilaterally (http://marsbar.sourceforge.net/, automated anatomical labeling Atlas, AAL [45]): amygdala for sucrose expectation (anxiety, anticipation), and dorsal anterior insula, ventral anterior insula, middle, medial and inferior orbitofrontal cortex (OFC), head of caudate nucleus, ventral striatum [46] and nucleus accumbens [47] for expected sucrose and artificial saliva receipt (taste and reward circuitry) [23].

### Statistical analysis

SPSS 28 software was used for statistical analyses (IBM, Armonk, N.Y.). Demographic and behavior data were analyzed using MANOVA. MANOVA and correlation analyses within HC or ED groups were used to test effects of potential confounding categorical or continuous variables such as comorbidity, medication use, BMI or age. Group-comparison studies were conducted with and without potential confounding covariates in the group-comparison (MANOVA, or MANCOVA for estimated marginal means). Brain imaging results are frequently non-normally distributed, and an additional group-comparison analysis was conducted using rank transformed values (Supplementary Material). Partial η^2^ was calculated for effect size in addition to power calculations. Post hoc group comparisons were furthermore Bonferroni corrected.

Regression analyses tested associations between behavior and brain activation, and results were multiple comparisons controlled using false discovery rate (FDR) [48].

Moderator analysis (PROCESS, SPSS) was used to test the effects of anxiety on the relationship between sucrose amygdala expectation response (X) and reward circuitry taste receipt response (Y). The primary hypothesis was that higher anxiety would moderate brain response to sucrose receipt. Those results were also FDR corrected for multiple comparisons.

## Results

### Demographic and behavioral variables

Demographic and behavioral variables are shown in Table 1. The overall age range was narrow with all mean values between 22 and 29 years of age, but AN and OSFED groups were younger than HC participants. AN was lower and BED higher in BMI compared to the HC group. Regular menses occurred in 16 AN (18%, 15 ± 7 days form last cycle), 17 OSFEDr (50%, 16 ± 8 days), all HC (6 ± 3 days), 33 BN (59%, 12 ± 8 days) and 6 BED (38%, 10 ± 6 days). Of the HC participants, 1 was American Indian/Alaska Native (0.8%), 13 were Asian (10.8%), 6 were Black or African American (5.0%), 3 were Asian/White (2.5%), 97 were White (80.8%); in the ED sample, 1 was American Indian/Alaska Native (0.5%), 2 were Asian (1.0%), 6 were Black or African American (3%), 2 were Asian/White (1.0%), 3 were Black, African American/White (1.5%), 1 did not identify race (0.5%), 1 was White/American Indian/Black, African American (0.5%), 181 were White (91.9%).

**Table 1:** Demographic variables.

	HC (A)		AN (B)		OSFEDr (C)		BN (D)		BED (E)		MANOVA analysis			
	(*n* = 120)		(*n* = 91)		(*n* = 34)		(*n* = 56)		(*n* = 16)					
	**Mean**	**SD**	**Mean**	**SD**	**Mean**	**SD**	**Mean**	**SD**	**Mean**	**SD**	**Effect Size (partial** ***η***^2^)	**F**	***p***	**Post hoc comparison**
Age (years)	25.15	4.95	21.85	5.82	22.10	5.92	23.52	4.65	28.60	7.39	0.10	9.10	<0.001	A, E > B***; A > C*; E > C***; E > D*
BMI (kg/m^2^)	21.49	1.64	16.39	1.05	20.66	3.09	23.21	7.06	32.92	9.52	0.67	159.50	<0.001	A, C, D, E > B***; D > C**; E > A, C, D***
TCI Harm Avoidance	10.74	5.23	21.85	7.63	23.94	6.86	23.96	6.49	17.56	7.23	0.40	52.50	<0.001	B, C, D > A***; E > A**; C, D > E*
STAI Trait Anxiety	27.35	5.65	56.20	11.97	59.29	11.26	61.05	11.27	46.31	13.80	0.58	105.40	<0.001	B, C, D, E > A***; C > E*; D > E***
STAI State anxiety	25.95	6.35	55.16	12.35	58.38	12.75	55.46	13.08	42.20	14.83	0.57	100.86	<0.001	B, C, D, E > A***; B, D > E*; C > E**
BIS/BAS Fight Flight Freezing	7.61	1.80	9.23	1.81	10.18	1.60	9.70	2.19	8.69	2.02	0.21	21.21	<0.001	B, C, D > A***
BIS/BAS-Anxiety	12.22	2.25	14.42	1.89	15.29	1.22	14.89	1.53	13.06	2.84	0.28	30.86	<0.001	C > B*; B, C, D > A***
BIS/BAS-Reward Responsive	17.72	1.81	16.63	2.31	16.62	3.03	16.25	2.55	17.25	2.21	0.07	5.44	<0.001	A > B, D**
BIS/BAS-Drive	10.58	2.08	10.34	2.76	9.94	2.58	10.48	3.12	10.50	1.93	0.01	0.45	0.773	
BIS/BAS-Fun Seeking	11.83	2.27	10.87	2.73	10.06	2.58	11.20	2.77	12.56	2.13	0.06	5.03	0.001	A, E > C**
BDI Depression	1.64	2.07	27.65	11.81	32.27	11.90	28.75	11.36	17.00	12.33	0.63	128.20	<0.001	B, C, D, E > A***; B, D > E**; C > E***
EDI-3 Drive for Thinness	1.98	2.96	19.07	7.44	21.73	6.77	22.05	5.38	18.44	6.74	0.61	121.60	<0.001	B, C, D, E > A***
EDI-3 Bulimia	0.76	1.03	5.59	7.09	7.18	6.91	18.18	7.68	19.44	7.33	0.55	93.90	<0.001	B, C, D, E > A***; D, E > B, C***
EDI-3 Body Dissatisfaction	4.22	4.96	24.72	10.27	31.61	9.03	29.98	9.04	27.50	7.91	0.60	116.30	<0.001	B, C, D, E > A***; C > B***; D > B*
Sucrose Pleasantness	5.03	2.27	4.23	2.44	3.50	2.14	4.54	2.60	5.44	2.61	0.05	4.20	0.003	A > C**; E > C*
Sucrose Sweetness	7.98	0.87	8.19	0.98	7.56	1.46	8.29	0.89	8.19	0.98	0.04	3.20	0.014	B, D > C*
Binge Frequency (weekly)	0.00	0.00	1.80	7.26	0.25	1.09	14.50	15.69	4.38	2.51	0.62	96.50	<0.001	D > A, B, C, E***
Purge Frequency (weekly)	0.00	0.00	3.05	10.22	3.67	6.28	16.02	17.27	0.00	0.00	0.43	43.40	<0.001	D > A, B, C, E***
Breakfast Calories (kcal)	605	133	584	153	605	180	596	185	623	112	0.01	0.40	0.798	
	***N***	**%**	***N***	**%**	***N***	**%**	***N***	**%**	***N***	**%**		***χ***^2^	***P*** **value**	
Antidepressant use	0.00	0.00	44.00	48.40	24.00	70.60	33.00	58.90	7.00	43.80		6.10	0.106	
Antipsychotic use	0.00	0.00	14.00	15.40	6.00	17.60	7.00	12.50	0.00	0.00		3.30	0.351	
MDD	0.00	0.00	43.00	47.30	20.00	58.80	31.00	55.40	4.00	25.00		6.00	0.113	
OCD	0.00	0.00	10.00	11.00	8.00	23.50	8.00	14.30	2.00	12.50		3.20	0.357	
PTSD	0.00	0.00	17.00	18.70	12.00	35.30	19.00	33.90	4.00	25.00		5.80	0.120	
Anxiety Disorder	0.00	0.00	59.00	64.80	21.00	61.80	42.00	75.00	6.00	37.50		7.90	0.047	B > E*

*HC* healthy control women, *AN* anorexia nervosa, *OSFEDr* other specific feeding and EDs with food restriction, *BN* bulimia nervosa, *BED* binge ED, *TCI* Temperament and Character Inventory, *BIS/BAS* Behavior Inhibition System/Behavior Activation System, *BDI* Beck Depression Inventory, *EDI-3* Eating Disorder Inventory 3, *MDD* major depressive disorder, *OCD* obsessive compulsive disorder, *PTSD* posttraumatic stress disorder.

*p  < 0.05; **p < 0.01; ***p  < 0.001.

Harm avoidance, trait and state anxiety, drive for thinness, body dissatisfaction, bulimia, were higher across ED groups compared to HC; BIS FFFS and BIS Anxiety were higher in AN, OSFEDr and BN groups compared to HC; binge and purge frequency was higher in BN compared to the other study groups; calories consumed during breakfast were similar across groups.

Across EDs, 108 (55%) individuals were on an antidepressant, and 27 (14%) on an antipsychotic. Ninety-eight (50%) individuals had major depressive disorder (MDD), 28 (14%) obsessive compulsive disorder (OCD), 52 (26%) posttraumatic stress disorder (PTSD), and 78 (40%) had generalized anxiety disorder (GAD). Across ED groups, there were no significant differences for comorbidity or medication use, except for lower anxiety disorder rate in BED compared to BN.

### Brain imaging results

In both HC and ED groups, scanner and age showed significant effects for sucrose expectation and receipt. In addition, within the ED group, PTSD had a significant effect on sucrose expectation, and comorbid anxiety disorder on sucrose receipt. Group-comparison analyses were conducted with and without covariates. Contrary to results in our previous study on prediction error response and unexpected stimulus receipt or omission, BMI was not significantly correlated with any regional response to stimulus expectation or expected stimulus receipt in either of the study groups.

### Group by condition analysis, expectation, and receipt response

**Fig. 1:**
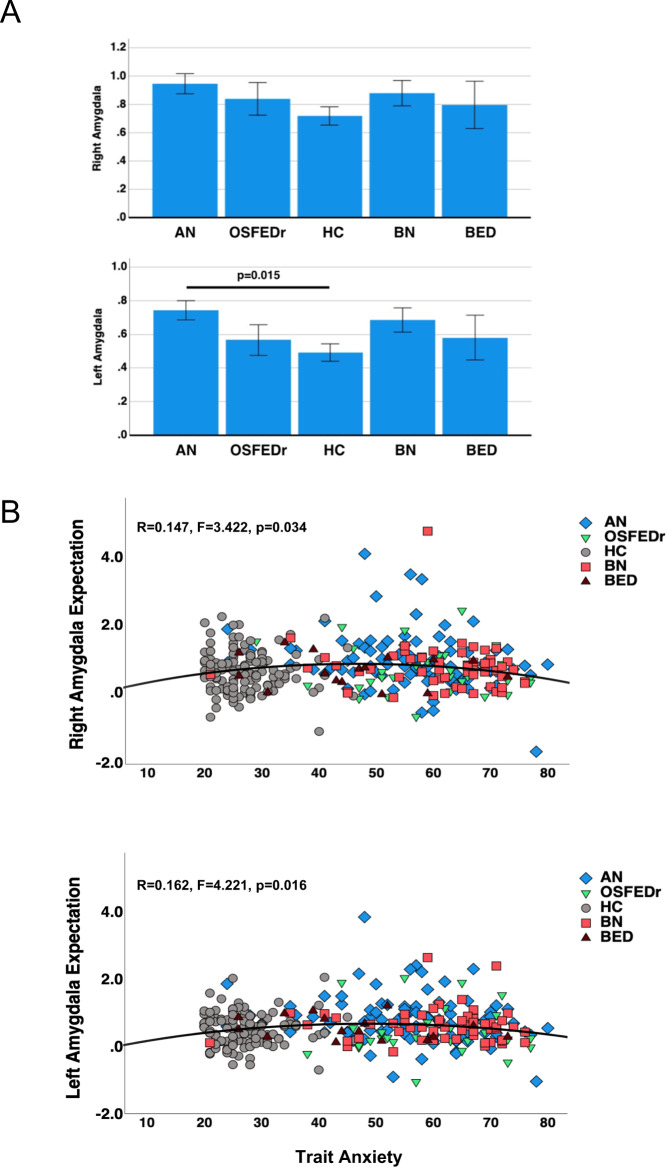
Across the study groups, left sided amygdala response to caloric taste stimulus expectation was significantly elevated in anorexia nervosa (AN) compared to healthy controls (HC), while activation in the remaining eating disorder groups only tended to be higher. In addition, moderately elevated trait anxiety was associated with stronger amygdala response, bilaterally, across the study sample. **A** Bar graphs for amygdala expectation response across study groups for estimated marginal means (5-group MANCOVA). **B** Correlation scatter plots for amygdala expectation response (beta values) and trait anxiety across the study sample; statistics are based on quadratic regression.

The 5-group by condition analysis for sucrose *expectation* (Table 2) showed an overall significant effect for group with Wilk’s lambda = 0.945, *p* = 0.023, a significant group effect (*F* = 4.08, *p* = 0.003) and post hoc analyses indicated higher left-sided amygdala response in the AN compared to the HC group (*p* = 0.002); the additional analysis with the covariates age, scanner, and PTSD showed similar results for left-sided group effect (*F* = 2.90, *p* = 0.022, Table 2, Fig. 1A), as did an analysis using rank transformed data (Supplementary Material).

**Table 2:** Amygdala sucrose expectation response.

	HC (A)				AN (B)				OSFEDr (C)				BN (D)				BED (E)				MANOVA analysis				
	(*n* = 120)				(*n* = 91)				(*n* = 34)				(*n* = 56)				(*n* = 16)								
	Mean	SE	LL 95% CI	UL 95% CI	Mean	SE	LL 95% CI	UL 95% CI	Mean	SE	LL 95% CI	UL 95% CI	Mean	SE	LL 95% CI	UL 95% CI	Mean	SE	LL 95% CI	UL 95% CI	Effect size (*η*^2^)	Power	F	*p*	Post hoc
Amygdala, Right	0.73	0.06	0.61	0.85	1.01	0.07	0.86	1.15	0.76	0.12	0.53	1.00	0.82	0.09	0.64	1.00	0.74	0.17	0.40	1.08	0.03	0.65	2.24	0.064	
Amygdala, Left	0.50	0.05	0.40	0.61	0.80	0.06	0.68	0.92	0.47	0.10	0.28	0.67	0.63	0.08	0.47	0.78	0.56	0.14	0.28	0.85	0.05	0.91	4.08	0.003	B > A, *p* = 0.002

	HC (A)				AN (B)				OSFEDr (C)				BN (D)				BED (E)				MANCOVA analysis (age, scanner, PTSD)				
	(*n* = 120)				(*n* = 91)				(*n* = 34)				(*n* = 56)				(*n* = 16)								
	EM mean	SE	LL 95% CI	UL 95% CI	EM mean	SE	LL 95% CI	UL 95% CI	EM mean	SE	LL 95% CI	UL 95% CI	EM mean	SE	LL 95% CI	UL 95% CI	EM mean	SE	LL 95% CI	UL 95% CI	Effect size (*η*^2^)	Power	F	*p*	post hoc
Amygdala, Right	0.72	0.06	0.59	0.85	0.95	0.07	0.81	1.09	0.84	0.12	0.61	1.07	0.88	0.09	0.70	1.06	0.80	0.17	0.47	1.13	0.02	0.43	1.38	0.242	
Amygdala, Left	0.49	0.05	0.39	0.59	0.74	0.06	0.63	0.86	0.57	0.09	0.38	0.75	0.69	0.07	0.54	0.83	0.58	0.13	0.32	0.85	0.04	0.78	2.90	0.022	B > A, *p* = 0.015

5-group MANOVA and MANCOVA analyses for extracted beta values, sucrose contrasted against non-caloric artificial saliva.

*EM* estimated marginal means, *p* values refer to the tests of between-subjects effects for group by region.

An additional analysis contrasting HC against the combined ED sample (Supplementary Material) showed higher amygdala response in the ED group compared to HC in the analysis without covariates (Wilks lambda = 0.979, *p* = 0.035, right amygdala ED > HC *p* = 0.049; left amygdala ED > HC *p* = 0.010), as well as in the analysis with covariates age, scanner and PTSD (Wilks lambda = 0.974, *p* = 0.017, right amygdala ED > HC *p* = 0.037; left amygdala ED > HC *p* = 0.005).

The 5-group by condition analysis for expected sucrose *receipt* was not significant for either MANOVA or MANCOVA (including age, scanner and generalized anxiety disorder as covariates; Supplementary Material), nor was an additional analysis contrasting the combined ED group against HC participants.

### Sucrose expectation brain response—demographic and behavior correlations

In the HC group, left amygdala response correlated positively with BIS-FFFS (*r* = 0.249, *p* = 0.006, CI 95% = 0.071 to CI 95% = 0.416).

In the ED group, amygdala response bilaterally correlated with age (R: *r* = −0.231, *p* = 0.001, CI 95% = −0.343 to CI 95% = −0.123; L: *r* = −0.205, *p* = 0.004, CI 95% = −0.314 to CI 95% = −0.091), body dissatisfaction (R: *r* = −0.193, *p* = 0.007, CI 95% = −0.324 to CI 95% = −0.068; L: *r* = −0.172, *p* = 0.017, CI 95% = −0.310 to CI 95% = −0.031), state anxiety (R: *r* = −0.162, *p* = 0.025, CI 95% = −0.286 to CI 95% = −0.042; L: *r* = −0.163, *p* = 0.025, CI 95% = −0.295 to CI 95% = −0.022), and right amygdala response with trait anxiety (*r* = −0.172, *p* = 0.017, CI 95% = −0.290 to CI 95% = −0.052), but not harm avoidance.

Across the entire study sample, there was a quadratic relationship between trait anxiety and bilateral amygdala expectation response (R: *r* = 0.147, *F* = 3.422, *p* = 0.034; L: *r* = 0.162, *F* = 4.221, *p* = 0.016) (Fig. 1B). Harm avoidance or BIS Anxiety showed no significant relationships with amygdala activation.

### Sucrose receipt brain response—demographic and behavior correlations

In the HC group, there were no significant correlations after multiple comparison correction (FDR).

**Fig. 2:**
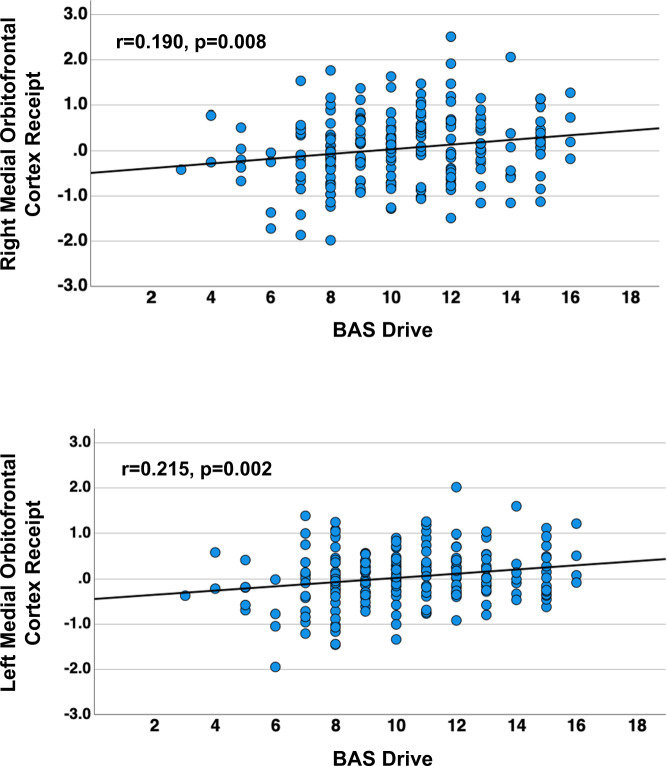
Within the eating disorder sample, Behavior Activation System (BAS) Drive was positively correlated with bilateral orbitofrontal cortex response to caloric taste stimulus receipt. Correlation scatter plots for medial orbitofrontal cortex expected sucrose taste receipt and BAS Drive across the eating disorder group.

In the ED group, BAS-Drive was positively correlated with bilateral medial orbitofrontal cortex (R: *r* = 0.190, *p* = 0.008, CI 95% = 0.064 to CI 95% = 0.311; L: *r* = 0.215, *p* = 0.002, CI 95% = 0.084 to CI 95% = 0.345) (Fig. 2), caudate head (R: *r* = 0.181, *p* = 0.011, CI 95% = 0.038 to CI 95% = 0.302; L: *r* = 0.187, *p* = 0.009, CI 95% = 0.054 to CI 95% = 0.309), ventral striatum (R: *r* = 0.200, *p* = 0.005, CI 95% = 0.074 to CI 95% = 0.313; L: *r* = 0.179, *p* = 0.012, CI 95% = 0.054 to CI 95% = 0.290) and left nucleus accumbens (*r* = 0.192, *p* = 0.007, CI 95% = 0.059 to CI 95% = 0.307).

### Correlations between sucrose expectation and expected sucrose receipt

Amygdala expectation response was significantly correlated with ipsilateral expected sucrose receipt response in ED groups across all (range *r* = 0.180 to 0.408, *p* = 0.008 to 0.000000003) and in HC across most regions (range *r* = 0.117 to 0.297, *p* = 0.202 to 0.0009) (Supplementary Material).

### Moderator analysis of anxiety on sucrose expectation–receipt interaction

In the HC group (Supplementary Material) no regional moderator analysis showed significant interactions after multiple comparison correction.

In the ED group, trait anxiety negatively moderated the relationship between expectation and receipt responses that remained significant after multiple comparison correction (FDR) for right amygdala sucrose expectation response and response to sucrose receipt in right dorsal anterior insula (*F* = 7.663, *p* = 0.006) and right ventral anterior insula (*F* = 8.21, *p* = 0.005) (Table 3). A test for subgroup effects in those regions indicated that in AN, there were significant moderator effects in the right dorsal anterior insula (*F* = 5.54, *p* = 0.021) and right ventral anterior insula (*F* = 6.37, *p* = 0.014), and in the BN group in the right ventral anterior insula (*F* = 4.88, *p* = 0.032).

**Table 3:** Moderator analysis for the interaction between trait anxiety and the relationship between amygdala expectation and reward circuit sucrose receipt response; interaction results remained significant for right dorsal and ventral anterior insular after FDR correction.

	Model summary			Model coefficients							Test of highest order unconditional interaction		
Region	*R*^2^	*F*	*P*		Coeff	se	t	*p*	LLCI	ULCI	*R*^2^ change	*F*	*p*
Dorsal Anterior Insula, Right [Sucrose Receipt Beta]	0.13	9.81	<0.001	[Right Amygdala Expectation Beta] Trait Anxiety Trait Anxiety Interaction	1.02	0.29	3.56	0.001	0.45	1.58	0.035	7.66	**0.006**
					0.02	0.01	2.83	0.005	0.00	0.03			
					−0.01	0.01	−2.77	0.006	−0.02	0.00			
Dorsal Anterior Insula, Left [Sucrose Receipt Beta]	0.16	12.02	<0.001	[Left Amygdala Expectation Beta] Trait Anxiety Trait Anxiety Interaction	0.97	0.28	3.47	0.001	0.42	1.52	0.027	6.15	0.014
					0.01	0.00	2.15	0.033	0.00	0.02			
					−0.01	0.00	−2.48	0.014	−0.02	0.00			
Ventral Anterior Insula, Right [Sucrose Receipt Beta]	0.11	8.23	<0.001	[Right Amygdala Expectation Beta] Trait Anxiety Trait Anxiety Interaction	1.46	0.41	3.54	0.001	0.65	2.27	0.038	8.21	**0.005**
					0.02	0.01	2.85	0.005	0.01	0.04			
					−0.02	0.01	−2.86	0.005	−0.03	−0.01			
Ventral Anterior Insula, Left [Sucrose Receipt Beta]	0.15	11.42	<0.001	[Left Amygdala Expectation Beta] Trait Anxiety Trait Anxiety Interaction	1.05	0.34	3.04	0.003	0.37	1.73	0.018	4.13	0.043
					0.01	0.01	1.69	0.093	0.00	0.02			
					−0.01	0.01	−2.03	0.043	−0.02	0.00			
Middle Orbitofrontal Cortex, Right [Sucrose Receipt Beta]	0.09	6.06	0.001	[Right Amygdala Expectation Beta] Trait Anxiety Trait Anxiety Interaction	0.10	0.22	0.46	0.650	−0.33	0.52	0.000	0.08	0.771
					0.00	0.00	0.69	0.489	−0.01	0.01			
					0.00	0.00	0.29	0.771	−0.01	0.01			
Middle Orbitofrontal Cortex, Left [Sucrose Receipt Beta]	0.13	9.88	<0.001	[Left Amygdala Expectation Beta] Trait Anxiety Trait Anxiety Interaction	0.38	0.29	1.32	0.189	−0.19	0.96	0.000	0.09	0.766
					0.00	0.00	0.22	0.823	−0.01	0.01			
					0.00	0.01	−0.30	0.766	−0.01	0.01			
Medial Orbitofrontal Cortex, Right [Sucrose Receipt Beta]	0.04	2.77	0.043	[Right Amygdala Expectation Beta] Trait Anxiety Trait Anxiety Interaction	0.73	0.41	1.77	0.078	−0.08	1.53	0.009	1.80	0.182
					0.01	0.01	1.37	0.172	0.00	0.03			
					−0.01	0.01	−1.34	0.182	−0.02	0.00			
Medial Orbitofrontal Cortex, Left [Sucrose Receipt Beta]	0.07	4.67	0.004	[Left Amygdala Expectation Beta] Trait Anxiety Trait Anxiety Interaction	0.59	0.34	1.72	0.087	−0.09	1.27	0.006	1.13	0.290
					0.00	0.01	0.82	0.412	−0.01	0.01			
					−0.01	0.01	−1.06	0.290	−0.02	0.01			
Inferior Orbitofrontal Cortex, Right [Sucrose Receipt Beta]	0.13	9.15	<0.001	[Right Amygdala Expectation Beta] Trait Anxiety Trait Anxiety Interaction	0.77	0.26	2.95	0.004	0.26	1.29	0.021	4.59	0.033
					0.01	0.00	2.67	0.008	0.00	0.02			
					−0.01	0.00	−2.14	0.033	−0.02	0.00			
Inferior Orbitofrontal Cortex, Left [Sucrose Receipt Beta]	0.15	11.73	<0.001	[Left Amygdala Expectation Beta] Trait Anxiety Trait Anxiety Interaction	0.67	0.29	2.31	0.022	0.10	1.24	0.007	1.53	0.218
					0.01	0.00	1.52	0.131	0.00	0.01			
					−0.01	0.01	−1.24	0.218	−0.02	0.00			
Caudate Head, Right [Sucrose Receipt Beta]	0.07	4.87	0.003	[Right Amygdala Expectation Beta] Trait Anxiety Trait Anxiety Interaction	0.86	0.36	2.43	0.016	0.16	1.57	0.017	3.48	0.064
					0.01	0.01	1.86	0.065	0.00	0.03			
					−0.01	0.01	−1.86	0.064	−0.02	0.00			
Caudate Head, Left [Sucrose Receipt Beta]	0.05	3.29	0.022	[Left Amygdala Expectation Beta] Trait Anxiety Trait Anxiety Interaction	0.63	0.39	1.60	0.112	−0.15	1.40	0.006	1.16	0.282
					0.00	0.01	0.50	0.618	−0.01	0.01			
					−0.01	0.01	−1.08	0.282	−0.02	0.01			
Ventral Striatum, Right [Sucrose Receipt Beta]	0.16	12.05	<0.001	[Right Amygdala Expectation Beta] Trait Anxiety Trait Anxiety Interaction	0.69	0.20	3.35	0.001	0.28	1.09	0.026	5.88	0.016
					0.01	0.00	1.95	0.052	0.00	0.01			
					−0.01	0.00	−2.42	0.016	−0.02	0.00			
Ventral Striatum, Left [Sucrose Receipt Beta]	0.18	14.18	<0.001	[Left Amygdala Expectation Beta] Trait Anxiety Trait Anxiety Interaction	0.69	0.22	3.09	0.002	0.25	1.14	0.017	3.89	0.050
					0.00	0.00	1.10	0.271	0.00	0.01			
					−0.01	0.00	−1.97	0.050	−0.02	0.00			
Nucleus Accumbens, Right [Sucrose Receipt Beta]	0.06	3.78	0.011	[Right Amygdala Expectation Beta] Trait Anxiety Trait Anxiety Interaction	0.77	0.33	2.37	0.019	0.13	1.41	0.019	3.79	0.053
					0.01	0.01	1.24	0.217	0.00	0.02			
					−0.01	0.01	−1.95	0.053	−0.02	0.00			
Nucleus Accumbens, Left [Sucrose Receipt Beta]	0.05	3.30	0.021	[Left Amygdala Expectation Beta] Trait Anxiety Trait Anxiety Interaction	0.49	0.32	1.53	0.127	−0.14	1.11	0.005	1.02	0.313
					0.00	0.00	0.40	0.689	−0.01	0.01			
					−0.01	0.01	−1.01	0.313	−0.02	0.01			

The bolded values indicate the values that remained significant after FDR correction.

An exploratory analysis of harm avoidance, depression scores, body dissatisfaction or drive for thinness did not show significant moderator effects in the HC or ED groups.

## Discussion

This study indicates that amygdala response is elevated in AN during expectation of caloric sweet taste stimuli compared to the HC group, while the other ED study groups only tended to have higher activation. Response to taste of the caloric stimulus was not different across groups. Amygdala caloric taste *expectation* and taste stimulus *receipt* response across taste reward regions were closely positively correlated, and across the ED sample, trait anxiety inversely moderated that relationship with the right insula. This result was confirmed in the smaller AN and partially in the BN subgroups. The study suggests that caloric sweet taste stimulus anticipation in individuals with EDs elicit a strong vigilance response, which drives reward circuit activation during taste stimulus receipt. However, trait anxiety diminishes that relationship, which could contribute to controlling food intake in individuals with EDs.

### Caloric stimulus amygdala expectation versus stimulus receipt response

Amygdala response to stimulus expectation was higher in the multivariate analysis in AN compared to HC, suggesting that expecting high caloric sucrose contrasted against non-caloric artificial saliva resulted in higher arousal. The response in the other ED groups tended to be higher but that was not significant after multiple comparison corrections. On the contrary, the expected sucrose receipt did not differ across groups. Neurobiological studies in the past have repeatedly associated amygdala response with negative emotionality and trait anxiety [16–18]. Individuals with EDs share not only the ED-specific behaviors drive for thinness, body dissatisfaction, and fear of weight gain, but also personality traits such as negative emotionality, perfectionism, and negative urgency [49–51]. High negative emotionality is characterized by a tendency to react with anxiety, fear, anger or sadness, and is associated with high trait anxiety [8–10, 52]. How negative emotionality including trait anxiety and ED behaviors interact neurobiologically is not well understood. This study indicates that caloric stimulus expectation elicits elevated amygdala response, especially in AN, which could be a state marker for negative emotionality toward the stimulus. The lack of group differences for stimulus receipt indicates that neural response to stimulus anticipation is more indicative of altered neurobiology in this group than the response to expected receipt.

### Caloric stimulus expectation predicts receipt response and is moderated by trait anxiety

Functional imaging in HCs had suggested that expectation biases neural response to stimulus receipt [36], while anticipatory anxiety has been shown to bias food intake in individuals with EDs [53]. The interaction of anxiety with the neural response to caloric stimulus expectation and receipt could provide a model for how anxiety affects ED-related neurobiology. Both the ED and HC groups showed very strong positive correlations between amygdala stimulus expectation activation and stimulus receipt response in taste reward-relevant regions. This supports previous studies linking expectation and receipt response and emphasizes that the amygdala is a key region for vigilance and anxiety processing that modulates cortical and subcortical regions that respond to taste receipt [31, 32]. Importantly, in the ED sample, trait anxiety moderated this relationship between the right amygdala and the right dorsal and ventral anterior insula. The insula is an important brain region for taste perception and body-related interoception and has strong connections with the striatal reward circuitry [54–56]. The right anterior insula has been specifically associated with self-recognition, the “abstract representation of oneself” and interoceptive awareness [57, 58]. It is, therefore, possible that high trait anxiety interferes with both normal taste and reward processing, as well as interoceptive awareness during food tasting. Trait anxiety could be an important link in ED pathophysiology by altering the normal taste expectation–receipt response.

### Trait anxiety and its relationship with amygdala response

Trait anxiety in the ED sample was negatively correlated with amygdala expectation response, which was significant on the right side. Across the whole study sample, a quadratic regression was the best fit for the trait anxiety–amygdala relationship, with an inverted-U-shaped curve that was significant bilaterally. Trait anxiety has been associated with ED psychopathology previously. Studies suggested that trait anxiety is related to low self-confidence and avoidance of social eating [14], and is associated with altered biological stress response across EDs [59]. Anxious traits have been also found to be important for altered interoception and anxious anticipation in AN [30]. It is therefore possible that anxious traits recruit amygdala-related circuitry and interfere with food approach [60].

An inverted-U pattern had been demonstrated previously for anxiety and arousal-related behaviors and underlying biological mechanisms [61, 62]. Whether very high levels of trait anxiety led to a desensitization of amygdala response in the ED sample perhaps because very high arousal levels are not sustainable requires further exploration. Harm avoidance was not significantly related to brain response in either study group, supporting previous studies that the underlying neurobiology of trait anxiety and harm avoidance differ [63]. State anxiety was also negatively related to amygdala expectation response; however, trait anxiety is more stable than state anxiety and we focused on the trait measure and its relationship with ED neurobiology.

### Behavioral approach system is related to caloric stimulus receipt response

Response to sucrose stimulus receipt in bilateral medial orbitofrontal cortex, caudate head, and ventral striatum in the ED group was significantly positively correlated with the BAS-Drive score. BAS-Drive reflects a person’s tendency to pursue rewards, a person’s “appetitive motivation”, and has been associated with cortical and subcortical response to food and non-food stimuli [40, 64, 65]. For instance, research that presented food pictures in the past found positive correlations with BAS-Drive score and orbitofrontal and ventral striatal activation [65]. The orbitofrontal cortex is an important region for reward valuation, and caudate and striatal regions process reward motivation. Individuals with EDs typically attempt to consciously control their food intake and it is possible that taste stimulation in this group is highly associated with the unconscious biological drive to pursue food reward, however, trait anxiety moderates that activation.

### Limitations

The study investigated a large transdiagnostic sample according to NIMH’s RDoC guidelines and group contrasts were analyzed for ED subgroups and the combined sample; however, OSFED and BED groups were small, and the restrictive OSFED and BED categories included different subgroups. A control group without ED psychopathology but with higher depression or anxiety scores could have further helped separate ED-specific versus comorbidity-driven brain response. All ED subgroups had higher amygdala response to caloric stimulus expectation but that was only significant in AN, while all participants with EDs had significantly higher trait anxiety and no group differed in sucrose receipt response. Effect size and power in the analyses were modest and larger groups may have identified significantly higher amygdala response also in other ED subgroups. BMI was not related to brain activation contrary to prediction error response previously, supporting that unexpectancy and thus dopamine related brain response is modulated by the amount of food intake; however, neural response to expected food stimulus receipt is not, suggesting different underlying neurotransmitter mechanisms. We assessed and controlled for potentially confounding effects of comorbid conditions, age or scanner; however, residual effects or type II errors cannot be excluded. Anxiety and depression are typically correlated but neither depression nor body dissatisfaction or drive for thinness moderated the expectation–receipt relationships. Here we focused on trait anxiety as a relatively stable measure [66]. Whether anxiety can be manipulated in an experiment and directly change taste expectation or receipt brain response will be focus of future studies. Most individuals with EDs who present to treatment are White and the results may not be applicable across all racial or ethnic groups. The ROI-based approach of this study was decidedly narrow to be in line with our previous studies. An exploratory whole brain analysis indicated clusters of higher activation across somatosensory, parietal, occipital and temporal cortex in the ED compared to HC group but those were not significant at the voxel level (Supplementary Material).

In summary, elevated amygdala response in AN and the combined ED sample suggests elevated arousal to food stimuli. The relationship between amygdala expectation and right insular stimulus receipt response is moderated by trait anxiety in individuals with EDs. The influence of trait anxiety on brain taste response supports the notion that anxious traits may interfere with normal reward and interoception processing and thus have an important role in perpetuating ED pathophysiology and psychopathology. The study raises the question whether modifying the effects of trait anxiety and associated arousal via psychopharmacologic or psychotherapeutic interventions could have an important role in facilitating ED-specific treatment.

## Supplementary information

Supplemental Material
