# Supplementary material for: Trait anxiety is associated with amygdala expectation and caloric taste receipt response across eating disorders
Source: Neuropsychopharmacology. 2022 Sep 13;48(2):380–90. doi: 10.1038/s41386-022-01440-z (PMC9750993; doi:10.1038/s41386-022-01440-z)
Supplement: Supplementary file 1 — Supplemental Material [file 41386_2022_1440_MOESM1_ESM.docx]

**Supplemental Material**

**S1. 3T GE Signa and Siemens Skyra 3T Scanner**

Brain imaging was performed between 0800 and 0900 hours on a 3T GE Signa or a Siemens Skyra 3T scanner: AN (GE Signa n=52, Siemens Skyra n=39), OSFEDr (GE Signa n=10, Siemens Skyra n=24), HC (GE Signa n=54, Siemens Skyra n=66), BN (GE Signa n=23, Siemens Skyra n=33) and 10 BED (GE Signa n=10, Siemens Skyra n=6), with a three-plane scout scan (16 seconds), sagittally acquired, spoiled gradient sequence T1-weighted (172 slices, thickness=1mm, TI=450ms, TR=8ms, TE=4ms, flip angle=12°, FOV=22cm, scan matrix=64×64), and T2*-weighted echo planar scans for blood-oxygen-level-dependent (BOLD) functional activity (3.4×3.4×2.6mm voxels, TR=2100ms, TE=30ms, flip angle=70°, 28 axial slices, thickness=2.6mm, gap=1.4mm). A Chi-square test indicated that healthy controls and individuals with eating disorders were similarly distributed across the two scanners (*χ*^2^=0.311, p=.58). Nevertheless, to account for potential scanner effects across groups, a scanner covariate was included in the MANCOVA model for imaging group contrasts.

Power calculations a priori were based on our previous prediction error data but applied to the amygdala. We extracted ROI based values for the bilateral amygdala for unranked values and calculated power and required sample size in SPSS (Power Analysis, One-Way ANOVA), using weighted cell size based on the current sample. Unranked prediction error values were for Right Amygdala AN M=9.4±11.5, OSFED M=7.7±9.1, HC M=6.3±5.1, BN M=7.0±5.5, BED M=4.4±2.8; and Left Amygdala AN M=6.8±10.3, OSFED M=6.0±5.3, HC M=4.2±2.9, BN M=5.5±5.7, BED M=3.4±2.7. The power analysis indicated for the Right Amygdala that a total of 277 subjects (AN=83, OSFED=28, HC=110, BN=42, BED=14) would be required for power of 0.8 to detect overall group differences (p<0.05, root-mean-square effect 0.274 in the small to medium range), with significant pairwise comparisons between AN and HC, and for the Left Amygdala a total sample of 257 (AN=77, OSFED=26, HC=102, BN=39, BED=13) for power 0.8, p<0.05 and small to medium effect size 0.256 (root-mean-square effect), to detect significant group differences, with significant pairwise comparisons for AN versus HC.

**S2. Taste Reward Task Paradigm**

The taste reward task design was adapted from O’Doherty et al. [1]. Participants received three taste stimuli during fMRI imaging (28 min. total task duration): 1 molar sucrose solution (100 trials), no solution (100 trials) and artificial saliva (80 trials). Participants learned to associate each unconditioned taste stimulus (US) with a paired conditioned visual stimulus (CS) that is probabilistically associated with its US: the CS shape for sucrose was followed in 80% of trials by sucrose solution (the other 20% were followed by no solution), and the CS shape associated with no-solution was followed in 80% of the trials by no solution (the other 20% were followed by sucrose); the CS shape for artificial saliva was always followed by saliva receipt. For each subject, the first 10 trials were fixed CS shape for sucrose followed by the delivery of US sucrose to establish an initial stable association between the CS sucrose shape and US sucrose taste [1]. All other trials were fully randomized without predetermined order. Taste stimuli were applied using a customized-programmable syringe pump (J-Kem Scientific, St Louis, MO, USA) and E-Prime Software (Psychological Software Tools, Pittsburgh, PA, USA) [2]. The MRI scanner radiofrequency pulse triggered taste application [3].

Study participants were compensated for their study participation with a total of $160, prorated for completion of questionnaires, diagnostic assessment and brain imaging scan.

**Figure S2.** The task design includes a Pavlovian learning model where participants learn to associate visual conditioned stimuli with unconditioned taste stimuli, sucrose solution, artificial saliva and no solution delivery. After the initial learning phase, participants will then receive taste stimuli expectedly or unexpectedly. For this study we analyzed only expected receipt trials.

**
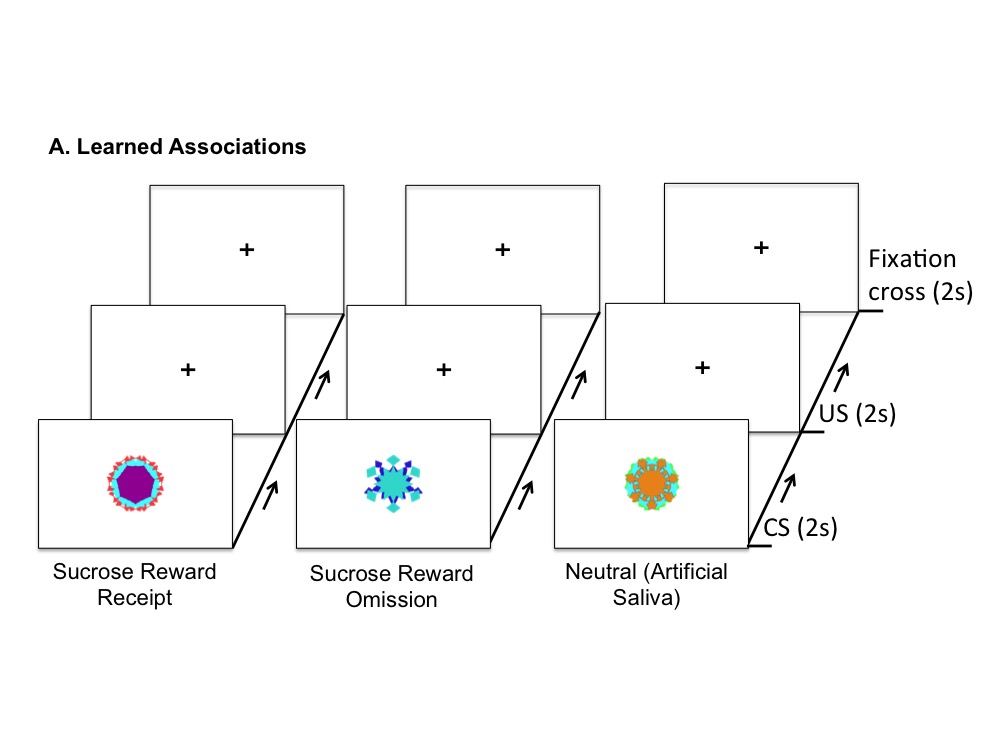

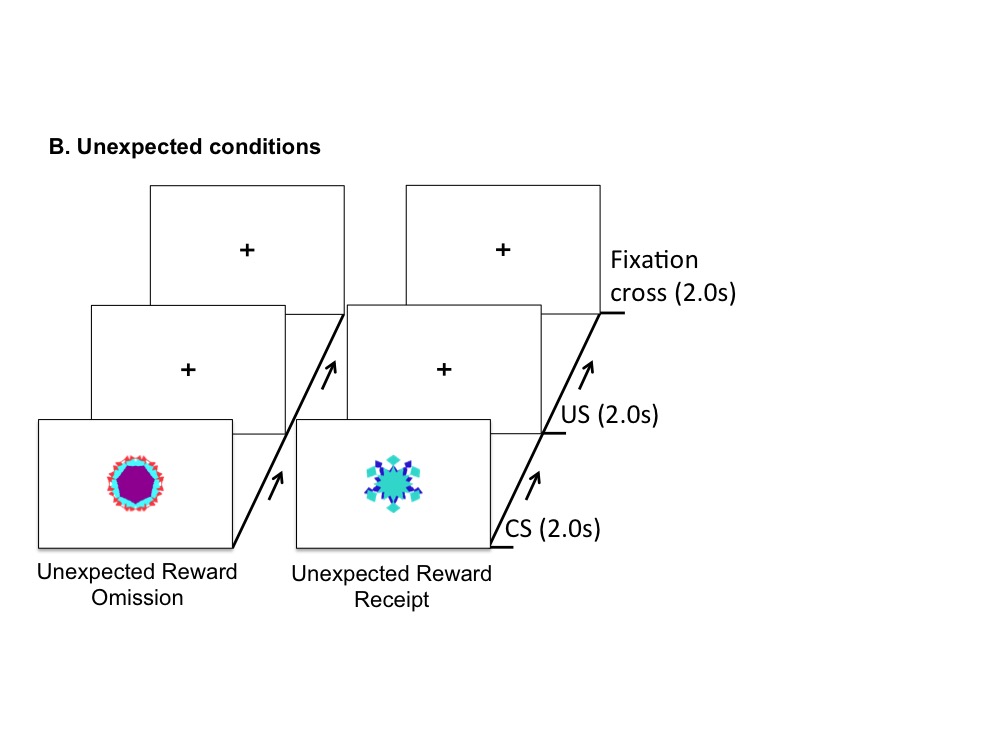
**

Panel A depicts the learned associations between the conditioned stimulus (CS, colored geometric shape, presented for 2 seconds (s)) and the unconditioned stimulus (US, 1ml sweet taste reward, presented for 2s). Intertrial interval was 6s. Panel B depicts the unexpected conditions where learned associations were violated during 20% of the trials. For this study, brain response to conditioned visual stimuli and stimulus receipt were modeled separately. Unexpected stimulus receipt or omission trials were not included in this analysis.

**S3. 5-Group comparison of Amygdala Expectation Response MANOVA Scatter Plots**

**S4. Rank transformed 5-Group comparison of Amygdala Expectation Response**

1. **Without covariates (MANOVA)**

1. **With covariates age, scanner and PTSD (MANCOVA)**

**S4. 2-Group Comparison of Amygdala Expectation Response**

1. **Without covariates (MANOVA)**

1. **With covariates age, scanner and PTSD (MANCOVA)**

**S5. 5-Group comparison of Taste Receipt**

1. **Without covariates (MANOVA)**

1. **With covariates age, scanner and GAD (MANCOVA)**

**S7. Correlation Results for Amygdala Expectation and Ipsilateral Taste Receipt**

1. **Healthy Controls**

1. **Eating Disorder Sample**

**S8. Moderator Analysis Trait Anxiety – Amygdala Taste Expectation, Reward Circuitry Taste Receipt for Healthy Controls**

**Exploratory Whole Brain Analysis**

We conducted an exploratory whole brain analysis across the combined eating disorder (ED) versus healthy control sample (HC), at p<0.001 and minimum 10 voxel contiguity for sucrose expectation parameter estimates. That analysis indicated no areas greater in HC compared to the ED group. The combined ED sample showed higher activation in cortical and subcortical areas compared to the HC group.

**References**

1 O'Doherty JP, Dayan P, Friston K, Critchley H, Dolan RJ. Temporal difference models and reward-related learning in the human brain. Neuron. 2003;38(2):329-37.

2 Frank G, Kaye W, Carter C, Brooks S, May C, Fissel K, et al. The evaluation of brain activity in response to taste stimuli--a pilot study and method for central taste activation as assessed by event related fMRI. J Neurosci Methods. 2003;131(1-2):99-105.

3 Frank GK, Reynolds JR, Shott ME, O'Reilly RC. Altered temporal difference learning in bulimia nervosa. Biol Psychiatry. 2011;70(8):728-35.
